# Development of a Low-Cost System for the Accurate Measurement of Structural Vibrations

**DOI:** 10.3390/s21186191

**Published:** 2021-09-15

**Authors:** Seyedmilad Komarizadehasl, Behnam Mobaraki, Haiying Ma, Jose-Antonio Lozano-Galant, Jose Turmo

**Affiliations:** 1 Department of Civil and Environment Engineering, Universitat Politècnica de Catalunya, BarcelonaTech. C/Jordi Girona 1-3, 08034 Barcelona, Spain; milad.komary@upc.edu (S.K.); Jose.turmo@upc.edu (J.T.); 2 Department of Civil Engineering, Universidad de Castilla-La Mancha, Av. Camilo Jose Cela s/n, 13071 Ciudad Real, Spain; Behnam.mobaraki@uclm.es (B.M.); Joseantonio.lozano@uclm.es (J.-A.L.-G.); 3 Department of Bridge Engineering, Tongji University, Shanghai 200092, China

**Keywords:** arduino, structural health monitoring (SHM), Internet of Things (IoT), accelerometer, low-cost sensors

## Abstract

Nowadays, engineers are widely using accelerometers to record the vibration of structures for structural verification purposes. The main obstacle for using these data acquisition systems is their high cost, which limits its use to unique structures with a relatively high structural health monitoring budget. In this paper, a Cost Hyper-Efficient Arduino Product (CHEAP) has been developed to accurately measure structural accelerations. CHEAP is a system that is composed of five low-cost accelerometers that are connected to an Arduino microcontroller as their data acquisition system. Test results show that CHEAP not only has a significantly lower price (14 times cheaper in the worst-case scenario) compared with other systems used for comparison but also shows better accuracy on low frequencies for low acceleration amplitudes. Moreover, the final output results of Fast Fourier Transformation (FFT) assessments showed a better observable resolution for CHEAP than the studied control systems.

## 1. Introduction

Civil structures and infrastructures could be considered as the main foundation of today’s modern society and, hence, their soundness is of utmost importance. However, the reports of ASCE infrastructure grades shows that in the United States: (1) 9.1% of all the bridges are not structurally efficient, (2) 188 million trips are taken every day over these deficient bridges, (3) The average age of bridges is 43 years old [1]. Monitoring and evaluating the health state of these structures are required for the maintenance applications, for minimizing the reparation costs and, eventually, for guaranteeing infrastructure safety [2,3,4]. Structural Health Monitoring (SHM) applications provide information on the state of structures, their functioning, and their structural response. As pointed out by many scholars (see, e.g., [5]), SHM can be used to calibrate structural models of real structures (digital twins [6]) that mimic the infrastructure performance to assess the decision-making process during the maintenance phase [7,8,9].

SHM systems are composed of sensors that measure the structural response (such as accelerations, rotations, strains, or deflections) over time. This information can be used to estimate changes in the structural performance of infrastructures [10,11,12]. The time variation of some environmental factors (such as temperature or humidity) that could produce crack opening, rotations, settlements, corrosion and other pathologies is so slow that they can be considered as quasi-static or static [13]. On the other hand, some events (such as the wave response due to earthquake ground motion, traffic-induced vibrations, or ambient activities) surely need to be accounted for the dynamic nature of the structural response they induce.

To observe and control them, dynamic SHM Systems are required [14,15,16]. Structural system identification is a critical component of SHM that targets to identify the parameters of the structural model [17]. Based on the nature of the structural response, structural system identification can be classified as static [18] or dynamic [19]. The static approaches have the advantage of being simpler and comparatively cheaper than the dynamic ones. However, tests are comparatively more difficult to perform, as some test loading is required that may lead to the closure to the service of the structure during testing. Moreover, to measure deflections, a frame of reference is needed, which is not always available. Hence, the paper targets the dynamic approach for the following reasons. (1) Dynamic approaches have been developed more actively. (2) Exciting a large structure dynamically or acquiring vibrations from natural dynamic excitation is easier than from static methods. (3) An internal reference frame for measuring accelerations is not required whereas it is for displacements [20]. A number of scholars (see, e.g., [21,22,23]) have presented different dynamic structural system identification techniques. These applications require some dynamic characteristics of the structure (such as frequencies or damping ratios), which could not be provided directly from the sensor responses [24]. To get the needed data for a Dynamic approach, the provided results from the accelerometers have to be analyzed by Operational Modal Analysis (OMA) methods [25]. Examples of these methods are the stochastic subspace method, peak picking (PP), or Frequency-Domain Decomposition (FDD). Most of the existing systems used to feed the dynamic modal analysis are equipped with commercial accelerometers [26,27].

Accelerometers are force-sensors attached to a seismic mass. When vibration is induced, this mass applies a specific force, which is proportional to the measured acceleration [28], and an electrical signal is obtained as a result. The most common type of vibration sensing technology is based on one of the following three main principles: piezoelectricity, piezoresistivity, and differential capacitive measurement [28]. Piezoelectric accelerometers use the piezoelectric effect of certain materials to measure dynamic changes in mechanical variables [29] and can operate on a wide range of frequencies [30]. The piezoresistive accelerometers (also known as strain gauge accelerometers) work by measuring the change in electrical resistance of a piezoresistive element when mechanical stresses are applied [31]. Differential capacitive accelerometers identify the displacement of the proof mass by measuring changes in their capacitance [31]. All these technologies for converting acceleration to an electrical signal (piezoelectric, piezoresistive, and capacitive change) could be combined to construct the last type of accelerometers, the micro-electro-mechanical systems (MEMS). These sensors are silicon-based micromachined devices that traditionally incorporate an accelerometer sensor and a signal conditioning circuitry [31]. The MEMS accelerometers have found their way to various industrial applications due to their significant on-going technology developments. Some of these accelerometers offer low-cost alternatives compared with traditional applications [32].

Information on different available accelerometers from various structural health monitoring applications is summarized in Table 1. This table has been ordered according to the price of the accelerometers.

The analysis of Table 1 shows a significant difference in sensor costs. The price of the most expensive sensor (3713B112G) is 385 times higher than that of the cheapest one (MPU6050). The cost of the accelerometers is precisely stated by scholars (see, e.g., [61]) as one of the main limitations for the practical application of SHM analyses. The price of acceleration acquisition methods is not limited to the accelerometers as they might include additional devices (such as real-time controller, data acquisition software, and workforce for data analysis). In this table, it can be seen that sensors with lower acceleration range (such as 4, 8) usually have lower noise density.

Arduino is a low-cost, easy to use, and open-source electronic prototyping platform which can be connected to the majority of analog, digital sensors. Moreover, the Arduino contains an Integrated Circuit Bus (I2C) and a Tx/Rx serial port for interfacing with sensors serially, making this microcontroller very flexible in interacting with various devices [62]. The main advantage of using this type of microcontroller is the fact that the Arduino platform and microcontroller rely on a very active developer and user community. This group is in continuous communication toward problem-solving. Moreover, it has a flexible design, a friendly interface and it is easy to learn. Finally, both open-source software and hardware of Arduino allow users to customize their devices [63]. In fact, many of the MEMS sensors can interact directly with an Arduino microcontroller [62]. Sensors 12 to 15 from Table 1 were the only low-cost MEMS accelerometers. They need an external power supply and could work with Arduino. As presented in Table 1, low-cost MEMS usually have higher noise density compared with the traditional commercial alternatives and do not offer a vast frequency range. As a result, their use in the literature was mostly dedicated to projects with strong motions and low frequencies [3,55,57,58,60,63] as they were not accurate enough to compete with traditional accelerometers on low acceleration ranges.

The literature review shows that no low-cost solutions are available to measure low accelerations with high accuracy that could be compared with traditional commercial sensors. To fill this gap, this paper develops a Cost Hyper-Efficient Arduino Product (CHEAP). This set is composed of five MPU9250 accelerometers controlled by an Arduino Due. The main novelty of this solution is its ability to increase the resolution and accuracy of the individual accelerometers by replicated measurements at the same time leading to a final averaged result with a higher measuring accuracy. To validate its performance on laboratory conditions, the CHEAP kit was compared with two piezoelectric sensors (393A03, 356B18) with low noise densities used as a control. In this test, dynamic movements with low range amplitudes and frequencies ranging from 0.5 to 10 Hz were tested. This test was done to compare the accuracy, resolution, and error of CHEAP with traditional expensive sensors. Although an acquisition system with 12 channels of 393A03 sensors is 14 times more expensive than an acquisition system with 12 sets of CHEAP, CHEAP works better on low frequency and low amplitude accelerations compared with 393A03.

This paper is organized as follows: Firstly, in Section 2, two piezoelectric sensors as the control systems are introduced together with their needed equipment. Then, CHEAP is fully explained and presented. Secondly, in Section 3, the laboratory test is used to validate the proposed methodology, and the obtained results are detailed. Finally, the main conclusions are drawn in Section 4.

## 2. Signal Acquisition and Processing System

In this section, the characteristics of CHEAP and control accelerometers are introduced. Moreover, the needed equipment for each sensor is reviewed together with their setting up protocol.

### 2.1. Control Systems Description

In this section, the main characteristics of the signal acquisition and processing system of two famous piezoelectric sensors are detailed. The acquisition equipment is presented as follows: (1) cRIO-9064: Embedded real-time sound and vibration input module controller that provides up to 12 channels [64], (2) NI9234, four-channel dynamic signal acquisition module that incorporates integrated electronic piezoelectric signal conditioner for accelerometers [65]. The needed power for the real-time controller was supplied through a constant current power supply. The signal conditioner, together with this power-supply, assured the constant current excitation to the sensors required for proper operation [49]. The program used for data acquisition was able to record the acceleration time-history from the two connected accelerometers simultaneously [49]. The bestowed program was created using NI LabVIEW 2016 [66].

Two individual piezoelectric accelerometers (393A03, 356B18) were connected to the introduced acquisition equipment for reporting separated readings. The sensor 393A03 was chosen for its low noise density. Consequently, it is used as a comparison benchmark for CHEAP. This sensor is a uniaxial piezoelectric accelerometer with a sensitivity of 1000 mV/g with a proof mass of 210 g [48]. On the other hand, the sensor 356B18 is a triaxial piezoelectric accelerometer that has the same sensitivity and a frequency range as low as 393A03 with a proof mass of 25 g [39]. Although the 356B18 has a higher noise density compared with 393A03, it was used as the second reference point for CHEAP. This second reference point was used because it was thought that CHEAP may not be able to provide data as accurate as 393A03. Although 393A03 has a noise density of 2 µg/√Hz, the accelerometers which are used to make CHEAP have each a noise density of 300 µg/√Hz. The rest of the characteristics of both sensors are listed in Table 1 (sensors 9 and 4, respectively).

**Figure 1:**
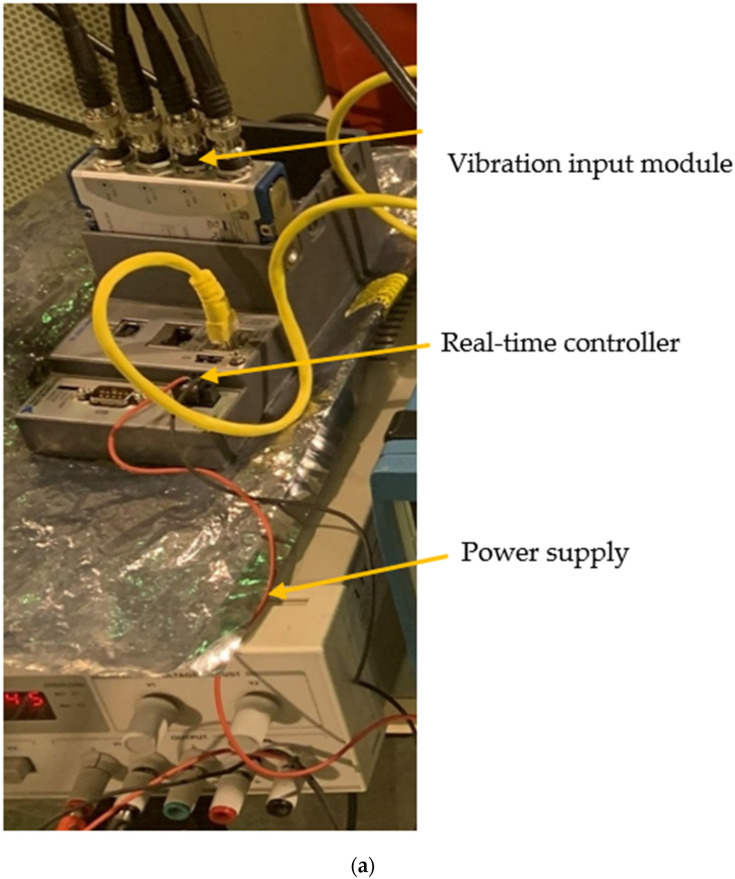

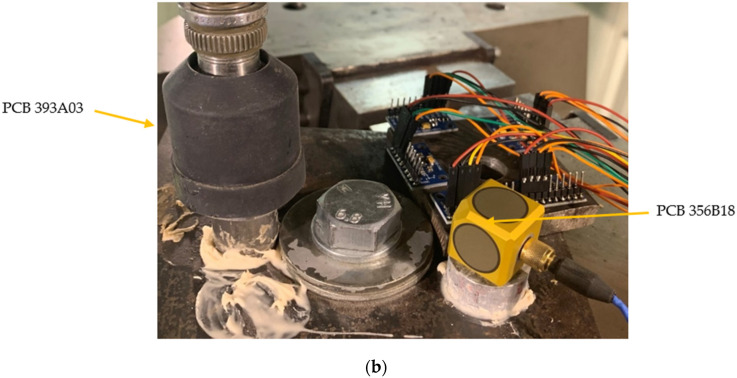
Control systems: (**a**) Data acquisition system for piezoelectric accelerometers and (**b**) Positioning of the accelerometers.

The acquisition system of the two studied accelerometers can be seen in Figure 1a. As illustrated in this figure, both accelerometers were connected to the real-time controller equipped with the vibration input module. Finally, the real-time controller was connected to a computer using a LAN wire. The used accelerometers and their positioning in the laboratory tests are illustrated in Figure 1b.

### 2.2. Cost Hyper-Efficient Arduino Product (CHEAP)

In this section, a low-cost system is proposed for the accurate measurement of accelerations. Instead of using the results of a single sensor, this approach averages the results of five similar low-cost MEMS accelerometers in order to amend the noises, improve the resolution, and lower the sensitivity of these factors. This number of sensors was finally selected by the experiences learned from the analyzed structures in the frame of the present research.

**Figure 2:**
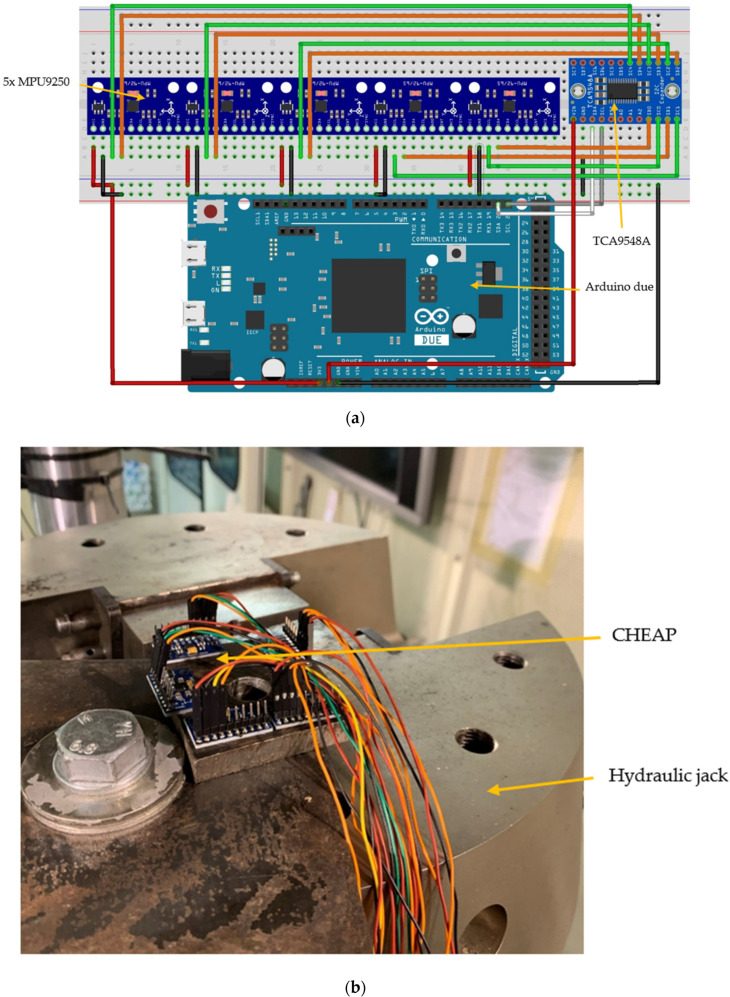
Developed signal and acquisition systems: (**a**) Schematic CHEAP and (**b**) CHEAP on the experiment jack.

CHEAP is composed of the following elements:

(1) Microcontroller: for this project, Arduino Due has been selected among many other options because, firstly, it can provide a reasonable amount of memory to upload complicated codes. Secondly, it has a faster clock speed (84 MHz) of communication compared with other alternatives. In Figure 2a, a sketch of this microcontroller created with the software Fritzing [67] is provided.

(2) Accelerometers: the reason why MPU9250 was chosen for CHEAP is the fact that this one is the newest among those that were presented in Table 1, has a reasonable price, uses less energy compared with MPU6050 with less noise density and has a better range of frequency in comparison with LIS344ALH and ADXL 335 especially on low-frequency signals.

(3) Multiplexor: MPU9250 uses the inter-integrated circuit (I2C) protocol for communicating with the Arduino [68]. I2C allows multiple “slave” digital integrated circuits (Sensors) to communicate with one or more “master” chips (Arduino). Each one of the sensors is introduced into the Arduino with a different address. On this application, five similar addressed MPU9250 have been used. Figure 2b shows the attachment of the low-cost accelerometers (MPU9250) on a stiff steel plate producing the sensing part of the CHEAP. The Arduino needs a different address for each connected component to its I2C port to interact and control the sensor. A multiplexer (TCA9548A) was used to change the address of similar sensors. The multiplexer has eight bi-directional switches that are controlled by the I2C bus. For introducing each sensor in the Arduino platform, only the address of this multiplexer and the occupied channel by the sensor on the multiplexer is required [69]. (4) Since CHEAP consists of five sensors, they have to be placed on a rigid plate. This plate should be from a material that would not absorb or dissipate the vibrations (such as steel or aluminum). The MPU9250 sensors have their *Z*-axis perpendicular to their surface. Since this paper presents a uniaxial sensor, all MPU9250 sensors must be glued to this plate with only their *Z*-axis paralleled with each other. (5) Connecting the system to the ground: the GND pin of Arduino Due must be connected to earth ground [70]. It was noticed that in the absence of this connection, the system initiation could face problems and rebooting the system would be required.

After the hardware set-up was finished, a code was written on the Arduino platform, which gets the acceleration from all five of the accelerometers (MPU9250) simultaneously. Experiences show that Arduino Due can print information with a frequency of 250 data per second (250 Hz) for one MPU9250. With more sensors connected to the Arduino, more data has to be printed by the microcontroller with the consequent speed reduction. In fact, the frequency decreases to 85 Hz when five of these MPU9250 sensors are connected. The data printing is a highly time-consuming operation, ergo the frequency of the overall kit decreases dramatically when more results have to be printed. The five sensors in CHEAP are not synchronized. The Arduino executes codes one line at a time. It means that when the code is executed, the Arduino connects with the first sensor and gets its measurement, and then, with the second one, and so on. This takes time. In the current CHEAP, the lag between each sensor-print is about 2.2 milliseconds. This lag is not hampering the FFT application, as this does not work with the exact time of data capture. However, if the timeline has to be improved, CHEAP measurement time output can be modified deducing 4.4 milliseconds (half the total lag between the first and last measurement).

Once recorded by Arduino, the data was saved into a PC using Python. This programming language was chosen because of its: (1) Connectivity: The library Serial enables a direct communication between Python and the Arduino serial-port, (2) Resolution: by using the date-time library, the exact capture time of data became possible with a resolution of one microsecond. To do so, Python saved the printed data from the Arduino serial port along with their capture-time on a text file. Finally, the acceleration from all five of the MPU9250 accelerometers was averaged and reported as the final output of CHEAP.

A few essential points need to be indicated about the CHEAP project: (1) Dependency: The python program needs to be run from a computer physically attached to the Arduino. In other words, the data acquisition equipment the present system needs is a computer. It is also important to mention that the used data acquisition equipment for commercial accelerometers (PCB 393A03 and 356B18) is also dependent on an attached computer. In a nutshell, both compared systems are not wireless, (2) Automation: Even though python can be scheduled for the experiments described in this paper it was activated manually. Since programming the jack for each experiment was time consuming, the beginning and finishing of the data collection for the commercial accelerometers as well as for the CHEAP were done manually, (3) Serial-port: The acquired data of both commercial accelerometers and CHEAP are transferred to the attached computer during the data acquisition, (4) Internet of Things: By running the written acquisition python code of CHEAP from a shared folder with OneDrive, the saved information was uploaded to cloud storage when the test was finished automatically. This way, the acquired data from every test is accessible.

**Figure 3:**
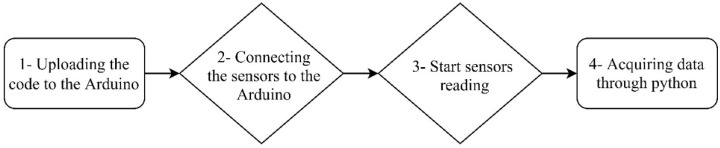
Diagram of needed steps for proposed kit data acquisition.

Figure 3 illustrates the required steps of the proposed metering system. This process is as follows: (1) Uploading the written code to the memory of the Arduino microcontroller from the Arduino platform. (2) Connecting all the sensors and the multiplexor to the Arduino. (3) Connecting the Arduino USB port to the computer activates the sensors. (4) Acquiring data by executing the code written in Python by the computer.

In a nutshell, CHEAP is an accelerometer with a low noise density and high resolution which is constructed from five low-cost accelerometers (MPU9250 sensor) with high noise density and consequently low resolution. Using a multiplexor, CHEAP receives data from all five accelerometers at the same time. At every time stamp, Arduino due receives the acquired data of all sensors. In the following, Arduino due averages the received data of five sensors and prints a single output which contains the inherent noises of the five MPU9250 sensors and the signals that are being studied.

CHEAP was developed for uniaxial data acquisition purposes, but MPU9250 has the possibility of recording data from all directions (Table 1). In other words, CHEAP is a potential triaxial accelerometer that has been programmed to be uniaxial. Unlike the usual uniaxial sensors, which only can acquire data only from one axis, CHEAP can be programmed to receive uniaxial data from any of the three directions. By programming three sets of CHEAP, one in the *X* direction, one in the *Y* direction, and one in the *Z* direction, a triaxial dynamic data acquisition system can be built.

It is also important to point out that the current accelerometer requires a voltage-current of 3.3 up to 5 Volt that consumes 200 mA per hour. Moreover, the currently developed accelerometer is not evaluated on an actual structure. However, for applying CHEAP for testing an actual structure, further developments are needed. CHEAP it is not waterproof or humidity-proof. For making this accelerometer waterproof, an appropriate box must be designed. Furthermore, CHEAP needs to be screwed or glued [71] properly [72] to the structure for accurate data acquisition.

## 3. Laboratory Test and Results

This section illustrates the resolution and accuracy of CHEAP in laboratory conditions. Firstly, the laboratory test performed is described. Then, the results obtained from the carried-out experiments are presented and discussed.

### 3.1. Laboratory Test

In this section, the equipment and test setup for producing the input acceleration time-waves are presented. In these tests, the acceleration recorded by the CHEAP were compared with those obtained by the control systems. These tests were carried out on the servo-hydraulic fatigue testing machine (INSTRON 8803 [73]) located at the Structural Laboratory Lluís Agulló of Technical University of Catalonia (Spain). This jack was programmed using WaveMatrix2 Dynamic Software [74].

To launch the acceleration time-wave signals, this device was programed to vertically move its lower jaw with various frequencies but with the same movement of ±0.1 mm from its equilibrium location. The input acceleration amplitudes for each frequency test was calculated by getting two time differential of the following movement equation:

(1) y=d×sin (2×π×f×t+φ),

where *y* represents the position of the lower jack plate based on the time *t*, *d* represents the maximum allowed Jack displacement (0.1 mm), *f* is the set frequency, and φ is the phase constant. By getting the second-order derivative of Equation (1), the accelerations presented in Equation (2) can be obtained.

(2) $$a=\frac{d^{2}\times y}{dt^{2}}=\ddot{y}=-d\times {\left(2\times \pi \times f\right)}^{2}\times \sin\left(2\times \pi \times f\times t+\varphi \right)$$

In total, 11 experiments were launched. Their characteristics are summarized in Table 2. In Table 2, the set frequencies of performed tests, input amplitude during each part of the test, and the number of performed cycles are presented. In this Table, IF is the Input Frequency, and IA is the Input Acceleration Amplitude. IF and IA are expected to be recorded by the accelerometers. In this table, the number of cycles was chosen to ensure that each experiment had the same data length for post-processing evaluations.

It is essential to mention that a real structure typically faces a sum of the waves introduced in Table 2. However, using FFT evaluation helps engineers to extract all the summed waves from a mixed signal and illustrate them individually. In fact, the commercial accelerometers are certified and calibrated on shaking tables and not on actual structures. The shaking table, which typically is uniaxial, induces vibrations within known acceleration amplitudes and frequencies. Furthermore, the results of the studied accelerometers are then compared with the known induced vibrations by the shaking table. The aforementioned information has been conducted through studying, communicating, and meeting with commercial companies. Such companies sell calibrated and certified accelerometers by testing their products on a uniaxial shaking table. Then, by changing the frequency and acceleration amplitude they validate the reliability of their products. Moreover, they recalibrate or certify custom made accelerometers such as CHEAP. In the future projects, CHEAP will be calibrated and certified in one commercial company to make a professional data-sheet for it. In the current work for avoiding high expenses of sensor certifications, the introduced experiments of this paper (Table 2) have been designed for frequency and acceleration accuracy tests.

The setting up of the sensors had to be done carefully in order to avoid any unwanted noise. Figure 1 and Figure 2 illustrate the set up for the test for both sensors (control and CHEAP). There are many ways of mounting the sensors; each one has its advantages and disadvantages, as reported in [71]. Since the surface flatness plays a vital role, special consideration was given to the mating surface. If needed, machining processes (such as lapping, spot-facing, grinding, milling, or turning) can provide an acceptably flat mounting surface [72]. In this work, for a proper attachment of the sensors, a steel plate was bolted to the jack firmly, and the sensors were glued to this plate to avoid independent vibrations.

The sampling frequency of the CHEAP kit was fixed on 85 Hz due to the speed capacity of Arduino. The sampling frequency for the two control systems was fixed to the same frequency for comparison purposes.

In this paper, after getting the saved signals from the accelerometers, they have been fed to the FFT assessment method.

While the control systems are feeding FFT with the data of each independent sensor, CHEAP uses averaged results of the five sensors to feed the FFT evaluation. The FFT process highlights the most captured signal as the main one and dials down the emphasis of the less frequent data. With this evaluation, the primary signal from the averaged data of five sensors gets more robust.

### 3.2. Results and Discussions

In this section, firstly, the frequencies and amplitudes obtained by the different sensors (CHEAP, 393A03, 356B18) are compared. Secondly, the Marginal benefits of increasing the number of sensors in CHEAP are studied. Finally, the price comparison of the different measuring systems is presented.

#### 3.2.1. Accuracy and Resolution of CHEAP

By comparing the final results of the CHEAP with those of the control systems acquired is studied in this section. Furthermore, the errors of their reported data from the input frequencies and acceleration amplitudes are reported in this section. Finally, the better functionality of CHEAP compared with the control sensors is shown.

After feeding the measured data from the accelerometers to the FFT application, frequencies and amplitudes of the experiment were calculated. In Table 3, frequencies extracted from each of the acquisition systems are presented together with their errors from the IF (Input Frequency). In this Table, MF is the Measured Frequency obtained by the sensors.

Table 3 shows that all the accelerometers work correctly on frequencies equal to and higher than 2 Hz and report the input frequencies precisely (IF). CHEAP (unlike the control systems) works well even for low range frequencies. In fact, while the control sensors were unable to allocate signals lower than 2 Hz, CHEAP was able to capture them. Although the data-sheet of the control systems (Table 1, sensor 9 and 4) illustrates that these sensors should be able to read frequencies from 0.5 Hz to 3000 Hz, the obtained results showed that they were not able to read accurately frequencies lower than 2 Hz with low acceleration amplitudes. The results of all applications are pretty close from 2 Hz to 10 Hz (less than 0.014% of error from the reference frequency).

In Table 4, amplitudes extracted from the accelerometers for the *Z*-axis, together with their errors from the IA (Input Acceleration), are presented. In this Table, MA refers to the Measured Acceleration amplitudes by the sensors.

The analysis of Table 4 shows that for those accelerations whose value was lower than 25.5, Mili-g CHEAP worked better than the 356B18. For the accelerations whose amplitude was lower than 14.5 Milli-g, CHEAP worked better than the 393A03. These data clearly show that CHEAP was able to compete with the two control systems. This Table also illustrates how the performance of CHEAP is especially interesting for small amplitudes.

In Figure, the MA error of the control systems and CHEAP is shown. The horizontal axis represents the frequency (Hz) of the experiment, and the vertical axis shows the error in percentage.

**Figure 4:**
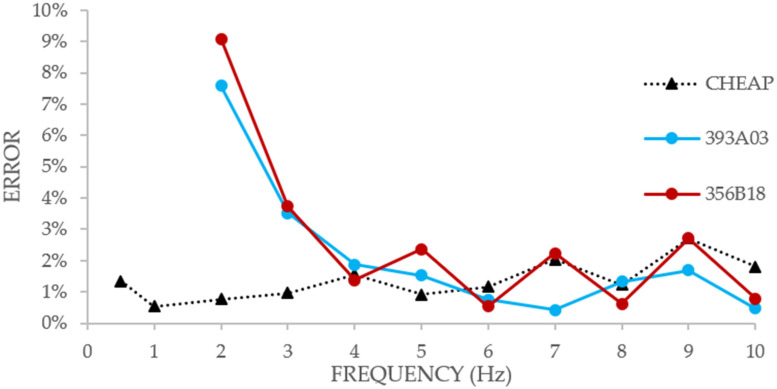
Comparing MA error of the two control systems with CHEAP.

Figure 4 shows that on lower amplitudes, CHEAP (compared with the two control systems) worked steadier and more accurately until 6 Hz. This figure also illustrates that the control systems only began to provide better accuracy on frequencies higher than 6 Hz. Moreover, in higher amplitudes, the errors of the different applications were quite close. The maximum experienced errors from the input acceleration amplitude on the highest experienced amplitude for CHEAP, 393A03 and 356B18 were as low as 0.47%, 0.79%, and 2.33%, respectively.

#### 3.2.2. Effect of the Number of Sensors

In this section, the beneficial effects of adding an increasing number of averaged sensors are studied in detail.

**Figure 5:**
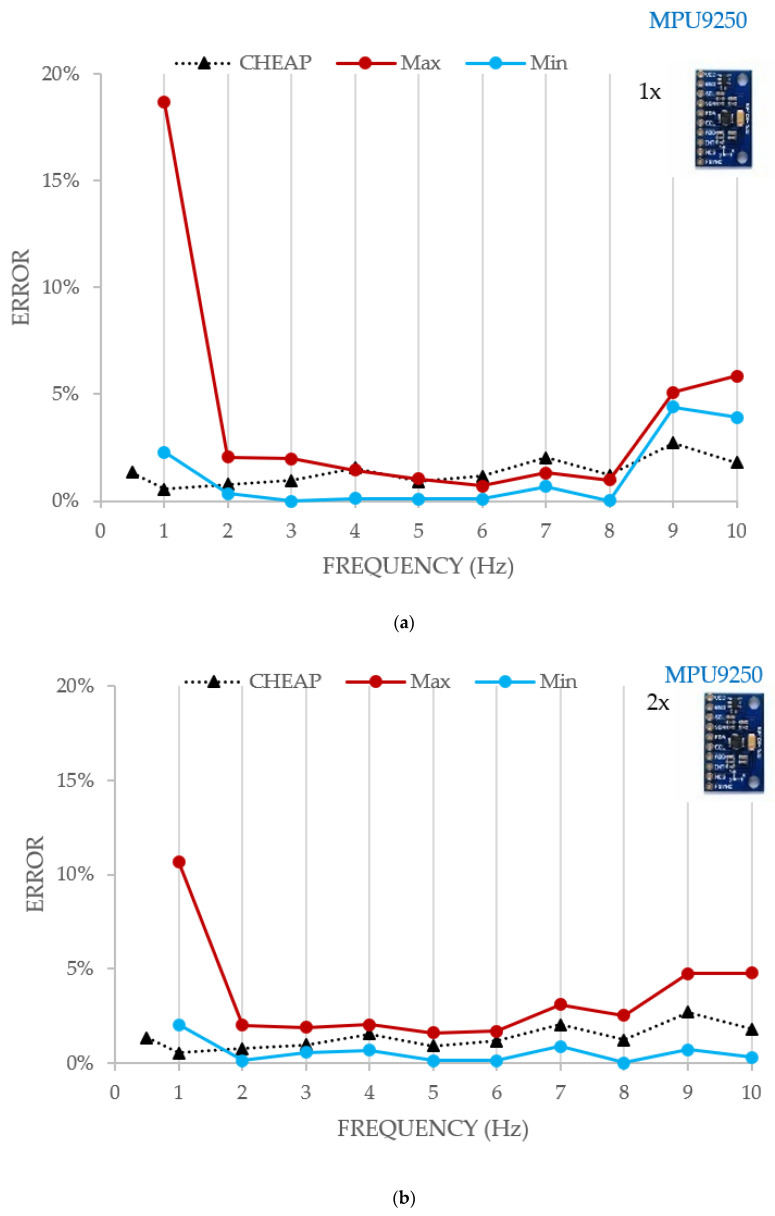

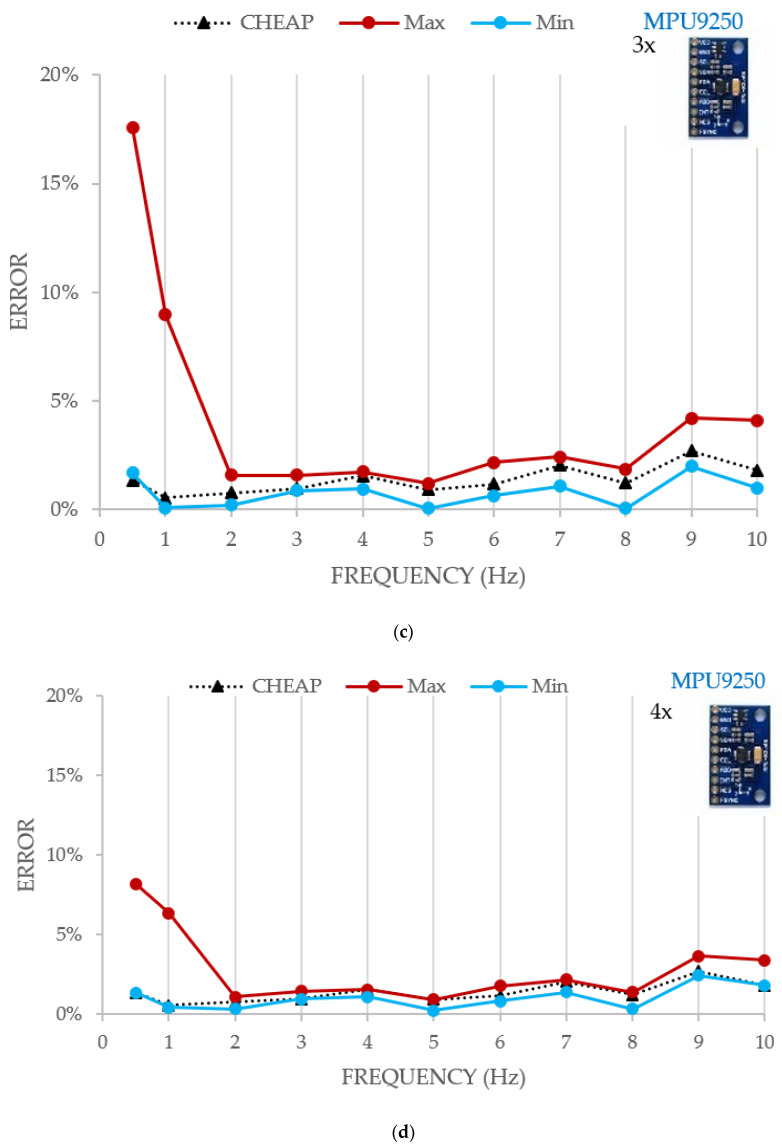
Estimated MA error for different number of sensors: one sensor (**a**), two sensors (**b**), three sensors (**c**), four sensors (**d**).

In Figure 5, estimated errors obtained for a different number of sensors in CHEAP are compared. The Max and the Min in each graph represent the enveloped error for all the possible sensor selections from the five available accelerometers (CHEAP represents the proposed kit with five sensors). It is also worth mentioning that in this work only the data outputs of *Z*-axis of MPU9250 sensors were used. Concordantly, the orientation of *X* and *Y* axes of these sensors are not important in this work. They have been distributed with only their *Z*-axis paralleled.

The results of the increasing number of sensors are presented in Figure 5, Figure 5a (one sensor), Figure 5b (two sensors), Figure 5c (three sensors), Figure 5d (four sensors). In all these figures, the horizontal axis presents the frequency of the experiment, and the vertical one illustrates the MA error in percentage. The MA for 0.5 Hz is not presented in Figure 5a,b because the system resolution for acquiring low acceleration amplitudes was insufficient. In other words, Figure 5a,b do not have the outputs of the test with the frequency of 0.5 Hz. The information of that test can be only analyzed and compared through Figure 5c,d. This is because the inherent noise of one or two accelerometers cannot be cancelled by FFT at 0.5 Hz. However, when three or more are used, noises can be canceled and some output for 0.5 Hz is obtained.

The analysis of Figure 5 shows that, as expected, the error depends to a greater extent on the number of sensors and the analyzed frequency. The errors of a system with one, two, three, four and five (CHEAP) sensors during the test with the frequency of 1 Hz were 18.67%, 10.68%, 8.95%, 6.35% and 1.45% respectively. Therefore, it can be concluded that lower errors are obtained when the number of accelerometers is increased, especially on the tests with lower acceleration amplitude (less than 0.4 milli-g). Results in Figure 5 also show that the part of the experiment which had the lowest frequency (0.5 Hz) could be considered as the most important one for the following reasons. Firstly, the highest experienced error appears there. Secondly, the lowest acceleration amplitude (0.1022 milli-g) is in this part of the experiment. In a nutshell, locating this low-level acceleration amplitude (MA) from the FFT evaluation was an opportunity to compare the resolution and accuracy of CHEAP with a different number of sensors. It should be highly noted that in order to locate the acceleration amplitude and frequency of an understudy signal from the FFT diagram, the noise density of the accelerometer must be smaller than the amplitude of the signals being studied. Consequently, the tests with one and two sensors did not have enough resolution. In other word their noise density was so high that the signal being studied could not be located from the FFT output diagram.

For a single MPU9250 accelerometer, the resolution for this part of the experiment was not enough. The resolution of the kit with a single accelerometer appeared to be at least 0.19 Milli-g. The amplitude of the needed signal was less than this resolution. As a result, finding and reporting this signal from the FFT output was not possible. The resolution for the kit of sensors with two MPU9250 accelerometers was not entirely clear either. This resolution was at least 0.13 milli-g, which is still 0.03 milli-g higher than the value of the captured signal. The resolution for the kit of sensors with three MPU9250 was about 0.10 milli-g. As a result, finding the amplitude of this part of the experiment was still impossible.

**Figure 6:**
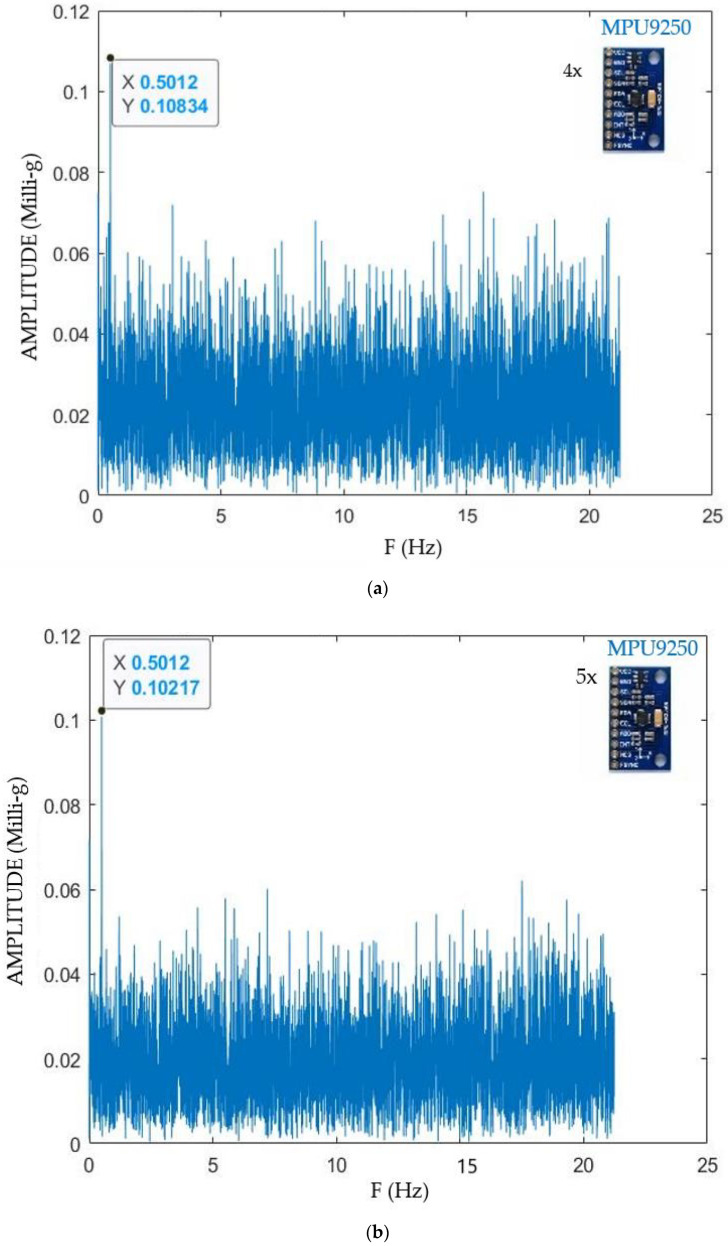
FFT data process for 0.5 Hz experiment errors for four sensors (**a**) and five sensors (**b**).

Results of the FFT application for the lowest tested frequency (0.5 Hz) for a different number of sensors is presented in Figure 6a (four sensors), Figure 6b (five sensors).

For reporting the resolution of each system from the FFT diagram, the amplitudes of acquired signals were investigated. It is known in this figure that the MA should have a frequency of 0.5 Hz. As a result, any other wave can be considered as an unwanted signal, and the highest amplitude among these unwanted signals is the resolution of this system. Table 4 reports the IA for the signal with a frequency of 0.5 Hz as 0.1006 Milli-g. The analysis of Figure 6a illustrates that with four MPU9250 accelerometers, the resolution of the system is slightly less than 0.08 Milli-g. This resolution enabled locating the needed signal from the FFT output diagram possible. This figure reports the MA of the signal as 0.10384 Milli-g, which has a 3.22% error from the IA. On the other hand, the analysis of Figure 6b shows that the kit of sensors with five MPU9250 (CHEAP) provides a resolution of around 0.06 Milli-g. In addition, it was deducted that CHEAP had an error of 1.55% from the IA.

A kit of sensors with five MPU9250 (CHEAP) has a sampling frequency of 85Hz and a resolution of 0.06 Milli-g. In addition, CHEAP provided exceptionally accurate outputs for accelerations less than 14.5 milli-g. Moreover, CHEAP worked properly where the commercial sensors were unable to provide any data whatsoever. As it was already discussed, the two studied control systems were not able to provide MA of the needed signal for the experiment with 0.5 Hz frequency. This was due to the low resolution of the control accelerometers. From the FFT outputs for the experiment with 0.5 Hz frequency, it was seen that 393A03 and 356B18 have a resolution of about 0.5 and 1.6 milli-g, respectively.

The reason why the averaged results of a number of accelerometers show a lower noise density and higher resolution lies within the processing procedure. During laboratory tests it was observed that all MPU9250 sensors have individual noises that changed from a test to another. Deep study of the FFT process has helped the authors to come up with a novel idea. By averaging the outputs of five MPU9250 sensors, a single output that includes the inherent noises of the accelerometers and the signals can be obtained. Therefore, the signals being studied are not dependent on the low-cost accelerometers, and they do not affect the FFT process. However, since the noise density of final product has a complete indirect relation with the number of averaged accelerometers, it is concluded that every single accelerometer has a unique noise density. The FFT highlights the most repeated signals (the signals being studied) and undervalues the signals that are repeated less such as the inherent noises of the accelerometers that have been repeated one-fifth of the number of times that the main signals have been repeated.

#### 3.2.3. Price Comparison

The overall price comparison of the used sensors and their equipment has been presented in this section.

In Table 5, the price of the equipment of each of the studied acquisition applications is presented. This Table includes the following information organized in columns: (1) System: the application which uses the illustrated accelerometer, (2) Price of the used accelerometers: the accelerometer of CHEAP was composed of five MPU9250 with a unitary cost of 5.76 €, a 3.22 € multiplexor (TCA9548A) and a 4.03 € breadboard, (3) Price of the microcontroller, (4) Price of the cable: the cable for all of the systems were three meters. CHEAP is using normal cables, whereas cables required by the two control systems are special noiseless cables, (5) Price of the real-time controller, (6) Price of the vibration input module, (7) Dimension of the sensing part, (8) Weight of the sensing part. It can be deducted that CHEAP is not much bigger or heavier than the control accelerometers. It has to be noted that although the used software for the control systems was 3549 €, CHEAP used the Arduino platform and Python, which are both free.

From the analysis of Table 5, it can be seen that the price of an acquisition system with a single 393A03 is 57 times higher than CHEAP. Nevertheless, the introduced equipment for the control systems has the capacity for more sensors. In order to make a fair price comparison between the control systems and CHEAP, the full capacity of the equipment should be taken into account. Real-time control provides 12 channels, and the vibration module has four channels. For a uniaxial control system on full capacity: 12 393A03 accelerometers, 12 sets of single channeled cables, one real-time controller, three vibration input modules are needed. The overall price is about 17,580 € (VAT excluded). For a triaxial control system on full capacity: four 356B18 accelerometers, four sets of three channeled cables, one real-time controller, three vibration input modules are needed. The overall price is about 14,200 € (VAT excluded). As it was mentioned before, three sets of CHEAP can be programmed to make a triaxial sensor. As a result of this potentiality, 12 sets of CHEAP can either be used as 12 uniaxial accelerometers or four triaxial accelerometers with a proximate price of 1008.84 (VAT excluded).

**Figure 7:**
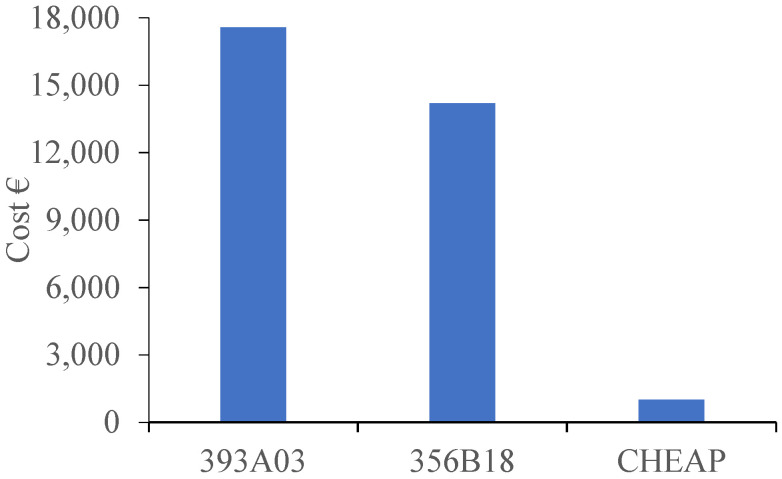
Price comparison of CHEAP with control systems.

In Figure 7, a comparison of the total price of the different measuring devices, when each acquisition system has 12 channels, is presented. This comparison does not take into account the price of the control system software nor the needed power supply for the control sensors.

As shown in Figure 7, the total price of an acquisition system with 12 channels of CHEAP is about 17 times lower than the control acquisition system with all the 12 uniaxial (393A03) accelerometers and 14 times lower than the same system occupied with four triaxial (356B18) accelerometers. Also, the needed equipment for running the CHEAP is fewer than the control systems, which would make setting up the CHEAP easier and faster than the control systems.

## 4. Conclusions

In order to determine maintenance applications, minimize the reparation costs, and guarantee the safety of the structures, Structural Health Monitoring (SHM) systems are required. SHM usually require costly sensors and equipment. This issue was a very significant drawback because the majority of structures were not economically eligible to go through SHM evaluations. To solve this issue, in this paper, a Cheap Hyper-Efficient Arduino Product (CHEAP) to record accelerations was introduced to decrease the cost of the SHM applications. CHEAP consists of five MEMS accelerometers (MPU 9250), a multiplexer, and an Arduino Due.

To validate the accuracy of CHEAP, its performance on laboratory conditions was compared with that of two control accelerometers (393A03, and 356B18). The main advantage of CHEAP in comparison with the commercial alternatives in the literature is its reduced cost. CHEAP is 58 times cheaper than an acquisition system with a single 393A03 accelerometer. For a fair comparison, all the available channels of the control acquisition system had to be used. These channels could be occupied either with 12 uniaxial accelerometers (393A03) or four triaxial accelerometers (356B18). It is known that 12 sets of CHEAP can both be used as 12 uniaxial sensors and four triaxial ones. It was concluded that an acquisition system with 12 online channels of CHEAP is 17 times cheaper than a control acquisition system with 12 sets of 393A03 and 14 times cheaper than four sets of 356B18.

CHEAP and the control systems worked accurately in the frequency range of 2 Hz until 10 Hz with no significant error from the input data. Although control systems were unable to capture frequencies below 2 Hz, CHEAP was able to work on frequencies as low as 0.5 Hz. CHEAP also showed a better resolution compared with the control systems. Besides, it was illustrated that CHEAP had better accuracy on low acceleration amplitudes. In addition, on accelerations lower than 14.5 milli-g, CHEAP worked more accurately than the 393A03 accelerometer. It is to be noted that until 25.5 milli-g CHEAP worked more accurately than the 356B18 accelerometer. The resolution of CHEAP was about 0.06 milli-g while it was noticed from FFT outputs that the resolutions of 393A03 and 356B18 were 0.5 and 1.6 milli-g, respectively.

Adding an increasing number of averaged sensors is investigated to have beneficial effects on the resolution and accuracy of CHEAP. It was seen on an experiment with a frequency of 0.5 Hz that a kit of sensors with four MPU 9250 had a resolution of about 0.08 Milli-g while with five accelerometers (CHEAP), the resolution was around 0.06 Milli-g.

In a nutshell, this developed application could be used for structural health monitoring of typical structures with a low budget; until now, monitoring them was not economical. CHEAP can also enable the accurate monitoring of infrastructures with low frequencies. Future research will aim to evaluate the performance of CHEAP in actual structures and to compare its behavior against other commercial systems. Checking the performance of cheap under higher frequencies and amplitudes is also envisaged.

## Figures and Tables

**Table 1:** Summary of the characteristics of the accelerometers commonly used in the literature.

Nº ^1^	Name ^2^	Price (€) ^3^	Acceleration Range (g) ^4^	Frequency Range (Hz) ^5^	Spectral Noise (µg/√Hz) ^6^	Operation Temperature (°C) ^7^	Structural Type ^8^	Type ^9^
1	3713B112G [33]	2070.0	±2.0	[0.00, 250]	22.90	[−54, +121]	Wind Turbine [34]	Tri, M
2	356B08 [35]	1610.0	±50.0	[0.50, 5000]	40.00	[−54, +77]	Bridge Crane [36]	Tri, P
3	356A45 [37]	1410.0	±50.0	[0.70, 7000]	125.00	[−54, +85]	Forward Swept Wing [38]	Tri, P
4	356B18 [39]	1300.0	±5.0	[0.50, 3000]	11.40	[−30, +77]	Motorbike Speedway Stadium [40]	Tri, P
5	KB12VD [41]	828.0	±0.6	[0.30, 2000]	0.06	[−20, +80]	Concrete School Building [42]	Uni, P
6	3711B1110G [43]	870.0	±10.0	[0.00, 1000]	107.90	[−54, +121]	Railroad Bridges [44]	Uni, M
7	KS48C [41]	750.0	±6.0	[0.25, 130]	0.60	[−20, +120]	Footway Bridge [45]	Uni, P
8	393B12 [46]	820.0	±0.5	[0.15, 1000]	1.30	[−54, +82]	Historical Masonry Structures [47]	Uni, P
9	393A03 [48]	710.0	±5.0	[0.50, 2000]	2.00	[−54, +121]	Brick Masonry Constituents [49]	Uni, P
10	352A24 [50]	540.0	±50.0	[1.00, 8000]	80.00	[−54, +121]	Hallow Square Beams [51]	Uni, P
11	352C33 [52]	380.0	±50.0	[0.50, 10,000]	39.00	[−54, +93]	Bridges [53]	Uni, P
12	ADXL335 [54]	10.7	±3.6	[0.50, 550]	300.00	[−40, +85]	Bridges [55]	Tri, M
13	LIS344ALH [56]	12.0	±2.0	[1.00, 500]	50.00	[−40, +85]	Steel beam [57]	Tri, M
14	MPU9250 [3]	5.8	±16.0	[0.24, 500]	300.00	[−40, +85]	Steel Pile and Column [58]	Tri, M
15	MPU6050 [59]	5.4	±16.0	[0.24, 500]	400.00	[−40, +85]	Building Model [60]	Tri, M

Notes: ^1^ Sensor number. ^2^ Sensor name. ^3^ Sensor price: the prices are obtained from retailers (VAT excluded). ^4^ Acceleration range: the maximum acceleration amplitude capacity of the sensors. ^5^ Frequency range: the accurate, readable range of frequencies. ^6^ Spectral Noise: the power spectral density of noise per unit of bandwidth (1 Hz). ^7^ Operational temperature: temperature range where the sensor works accurately. ^8^ Structural type: where the sensors are used. ^9^ Type: Uni stands for uniaxial, Tri for triaxial, P for piezoelectric and M for MEMS (uniaxial accelerometers are only capable of sensing vibration from one axis, while triaxial ones can sense vibrations from all of the directions).

**Table 2:** Characteristics of introduced Waves.

IF (Hz)	IA (Milli-g)	Number of Cycles
0.5	0.1006	200
1.0	0.4024	400
2.0	1.6097	800
3.0	3.6219	1200
4.0	6.4389	1600
5.0	10.0610	2000
6.0	14.4874	2400
7.0	19.7191	2800
8.0	25.7550	3200
9.0	32.5970	3600
10.0	42.9300	4000

**Table 3:** Frequency extracted from the acquired accelerometers together with their error.

IF (Hz)	393A03		356B18		CHEAP	
	MF (Hz)	Error (%)	MF (Hz)	Error (%)	MF (Hz)	Error (%)
0.5000	-	-	-	-	0.5012	0.2420%
1.0000	-	-	-	-	0.9993	0.0690%
2.0000	2.0003	0.0150%	2.0003	0.0150%	2.0002	0.0100%
3.0000	3.0005	0.0167%	3.0005	0.0167%	2.9996	0.0133%
4.0000	3.9997	0.0075%	3.9997	0.0075%	3.9996	0.0100%
5.0000	4.9998	0.0040%	4.9998	0.0040%	5.0007	0.0140%
6.0000	6.0002	0.0117%	6.0002	0.0117%	5.9997	0.0050%
7.0000	7.0004	0.0057%	7.0004	0.0057%	6.9994	0.0086%
8.0000	8.0006	0.0075%	8.0006	0.0075%	7.9991	0.0112%
9.0000	8.9998	0.0022%	8.9998	0.0022%	9.0004	0.0044%
10.0000	9.9996	0.0040%	9.9996	0.0040%	10.0004	0.0040%

**Table 4:** Amplitudes extracted from the accelerometers together with their error.

IA (Input Wave) (Milli-g)	393A03		356B18		CHEAP	
	MA (Milli-g)	Error (%)	MA (Milli-g)	Error (%)	MA (Milli-g)	Error (%)
0.1006	-	-	-	-	0.1022	1.5530%
0.4024	-	-	-	-	0.3966	1.4538%
1.6097	1.7319	7.5900%	1.7561	9.0934%	1.5977	0.7468%
3.6219	3.4947	3.5113%	3.7569	3.7281%	3.6638	1.1576%
6.4389	6.3189	1.8635%	6.3507	1.3696%	6.3536	1.3245%
10.0608	9.9082	1.5164%	10.2988	2.3660%	10.0016	0.5880%
14.4918	14.5964	0.7517%	14.4106	0.5308%	14.5063	0.1298%
19.7191	19.8035	0.4281%	20.1582	2.2268%	19.3468	1.8880%
25.7555	25.4122	1.3331%	25.9151	0.6195%	25.5072	0.9642%
32.5969	33.1459	1.6843%	33.4779	2.7028%	33.5534	2.9345%
40.2430	40.0529	0.4725%	40.5612	0.7906%	41.1806	2.3298%

**Table 5:** Price comparison of the three systems.

System	Cost of the Accelerometer (€)	Cost of the Microcontroller (€)	Cost of the Cable (€)	Cost Real-Time Controller (€)	Cost Vibration Input Module (€)	Dimension of the Sensing Part (mm)	Weight of the Sensing Part (gr)
393A03	710	-	75	2010	2050	28 × 28 × 56	210
356B18	1300	-	210	2010	2050	20 × 26 × 20	25
CHEAP	36.1	38.017	10	-	-	50 × 50 × 10	357

## Data Availability

Data is contained within the article.
